# Earliest Animal Cranial Surgery: from Cow to Man in the Neolithic

**DOI:** 10.1038/s41598-018-23914-1

**Published:** 2018-04-19

**Authors:** Fernando Ramirez Rozzi, Alain Froment

**Affiliations:** 1 AMIS UMR 5288 CNRS. Faculté de Chirurgie Dentaire, 1 rue Maurice Arnoux, 92120 Montrouge, France; 2 IRD - Musée de l’Homme, 17 place du Trocadéro, 75116 Paris, France

## Abstract

The earliest cranial surgery (trepanation) has been attested since the Mesolithic period. The meaning of such a practice remains elusive but it is evident that, even in prehistoric times, humans from this period and from the Neolithic period had already achieved a high degree of mastery of surgical techniques practiced on bones. How such mastery was acquired in prehistoric societies remains an open question. The analysis of an almost complete cow cranium found in the Neolithic site of Champ-Durand (France) (3400-3000 BC) presenting a hole in the right frontal bone reveals that this cranium underwent cranial surgery using the same techniques as those used on human crania. If bone surgery on the cow cranium was performed in order to save the animal, Champ-Durant would provide the earliest evidence of veterinary surgical practice. Alternatively, the evidence of surgery on this cranium can also suggest that Neolithic people practiced on domestic animals in order to perfect the technique before applying it to humans.

## Introduction

Evidence of cranial surgery in human history exists as early as the Mesolithic period before spreading even further during the Neolithic^1–3^. It is well represented throughout the world, and its use has been documented in skeletal remains from every continent; most of them from the Mesolithic to the present time^2–17^ (http://www.holeintheheadmovie.com). The most ancient example of a trepanation is described by Samuel George Morton^18^, in his famous book *Crania Americana* published in 1839: a skull from South America displayed a hole that Morton did not recognize as a trepanation, but as a wound attributed to a blunt instrument. The purpose of such a practice to treat functional disorders or as part of a magical-religious ritual has long been discussed without arriving at a conclusive answer^14^. The purpose of such a practice, we suppose, most probably depends on the societies and/or the period in question. Independently of the reasons that led humans to carry out trepanations, one cannot but be amazed by prehistoric man’s knowledge and mastery of the techniques of cranial surgery. Indeed, the oldest crania with evidence of trepanation reveal the use of the same techniques as those used in historic times with the same degree of accuracy^19^. Similar techniques are recorded all over the world. The bone was scraped or cut or drilled preventing any break of the inner table of the skull bone so as not to compromise the health and integrity of the brain.

How people involved in this cranial surgery acquired the training to practice the operation on humans is unknown. It is possible that they practiced on the skulls of the dead, but in that case the gestures could not have been fully assessed. That is to say, gestures developed on the crania of cadavers could lead to brain damage when practiced on living patients and it would be difficult to recognize dangerous gestures on anyone but live patients. It is also possible that they trained on live animals. A wild boar cranium (*Sus scrofa*) probably from a Neolithic site in Roquefort, France shows signs of a surgical operation^20^. Unfortunately, complete skulls of animals are rarely found in archeological sites since they were eaten and the skulls were most probably broken to extract the tongue and the brain.

The Neolithic site of Champ-Durand, Vendée, France, situated at around 40 km from the Atlantic coast, on the northern border of the Poitevin marshes, was a fortified locality with three series of ditches and described as an important trade center for local populations specialized in salt production and trade as well as in cattle slaughter in 5000 BP^21,22^ (Supplementary Information). Archeological excavations of the ditches carried out from 1975 to 1985 enabled researchers to find important quantities of faunal remains. Cut-marks on bones and burned bones indicate that domestic animals such as cows, pigs, sheep and goats were the principal source of meat^23^. Similarly to other neighboring Neolithic sites, the cow (*Bos taurus*) is the species most represented and corresponds to 54% of animal remains. An almost complete cow cranium, lacking only the anterior part of the maxilla and the extremities of the horns, shows a hole in the right frontal bone. In a previous work, the hole was interpreted as resulting from goring by another cow^23^, however a quick visual inspection of the bone surface shows some features that seem to indicate that the hole may be related to human activity. The aim of this work is to assess if the hole in the cow cranium is the result of human intervention.

## Results

**Figure 1:**
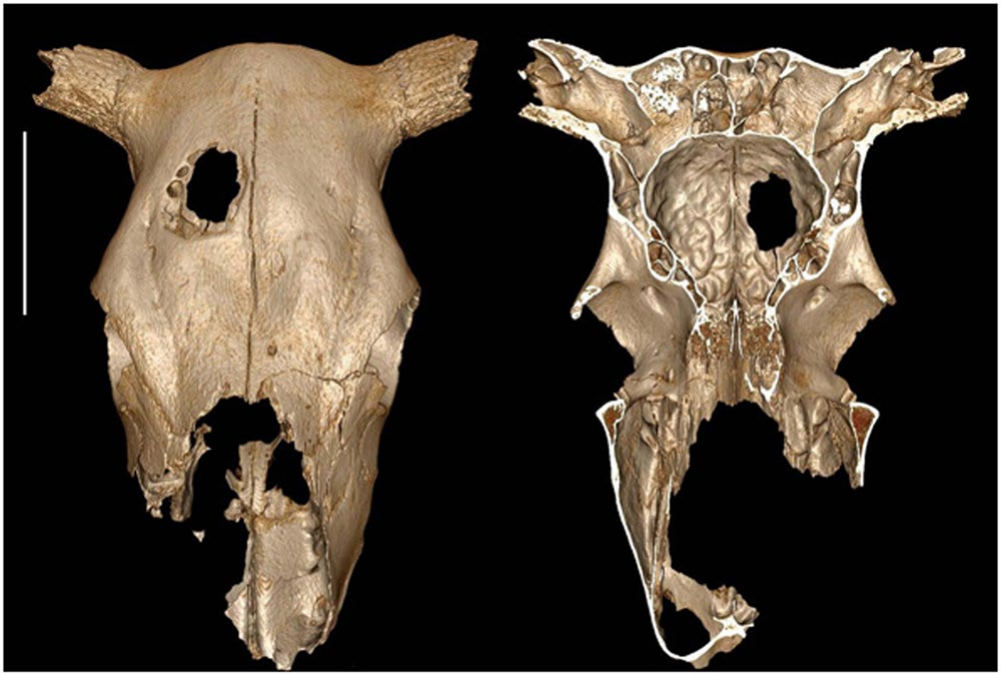
External and internal view of the cow cranium showing the hole on the right frontal bone. Bar corresponds to 10 cm.

The hole shows an anterior-posterior orientation measuring 64.5 mm long and 46.5 mm wide at the outer table (Fig. 1). It becomes smaller internally with a length of 40 mm and a width of 30 mm at the inner table. Change in size results from the fact that the outer table was more extensively removed than the inner table in the front area of the hole. The borders of inner and outer tables are irregular indicating that the bone was not cut. There is no other sign of trauma on any part of this cranium. There is a complete lack of evidence to support a violent origin for this trauma such as goring by another cow. A blow causing an injury of this shape would need to be struck almost perpendicularly to the bone surface. It seems reasonable to suggest that such a blow would produce fracturing either in stellate or comminuted form in and around the wound. No evidence of such a fracture, either internally or externally can be seen. Furthermore, if the hole was produced by a shock, internally orientated bone splinters should be observed. However, 3-D reconstruction from X-scan projections and SEM analyses fail to show internally orientated splinters but does show a continuous surface of endocranium all around the hole (Supplementary Fig. 1). The bone surfaces are not smooth, the edges of the hole are sharp and the diploeic pores are easily visible. Radiological studies confirm the lack of any healing processes in the bone tissue around the hole. Therefore the animal did not survive the injury or was killed shortly afterwards or the trauma occurred once the animal was already dead.

Holes in skull bones can also be related to infectious diseases, like syphilis or tuberculosis, local benign or malignant tumors, metastases, congenital defects, or taphonomic events like the action of gnawing animals, insects, or selective erosion. In our case the regularity of the hole, and the lack of periostal inflammation, does not favor an origin such as a tumoral or infectious lesion, unless the margins of the initial lesion had been cut out and removed during the surgical process. Internal orifices accompanying areas of trepanation have a regular appearance: they are smooth and seem to correspond to the pneumatization of the sinuses or other cavities in a normal skull.

**Figure 2:**
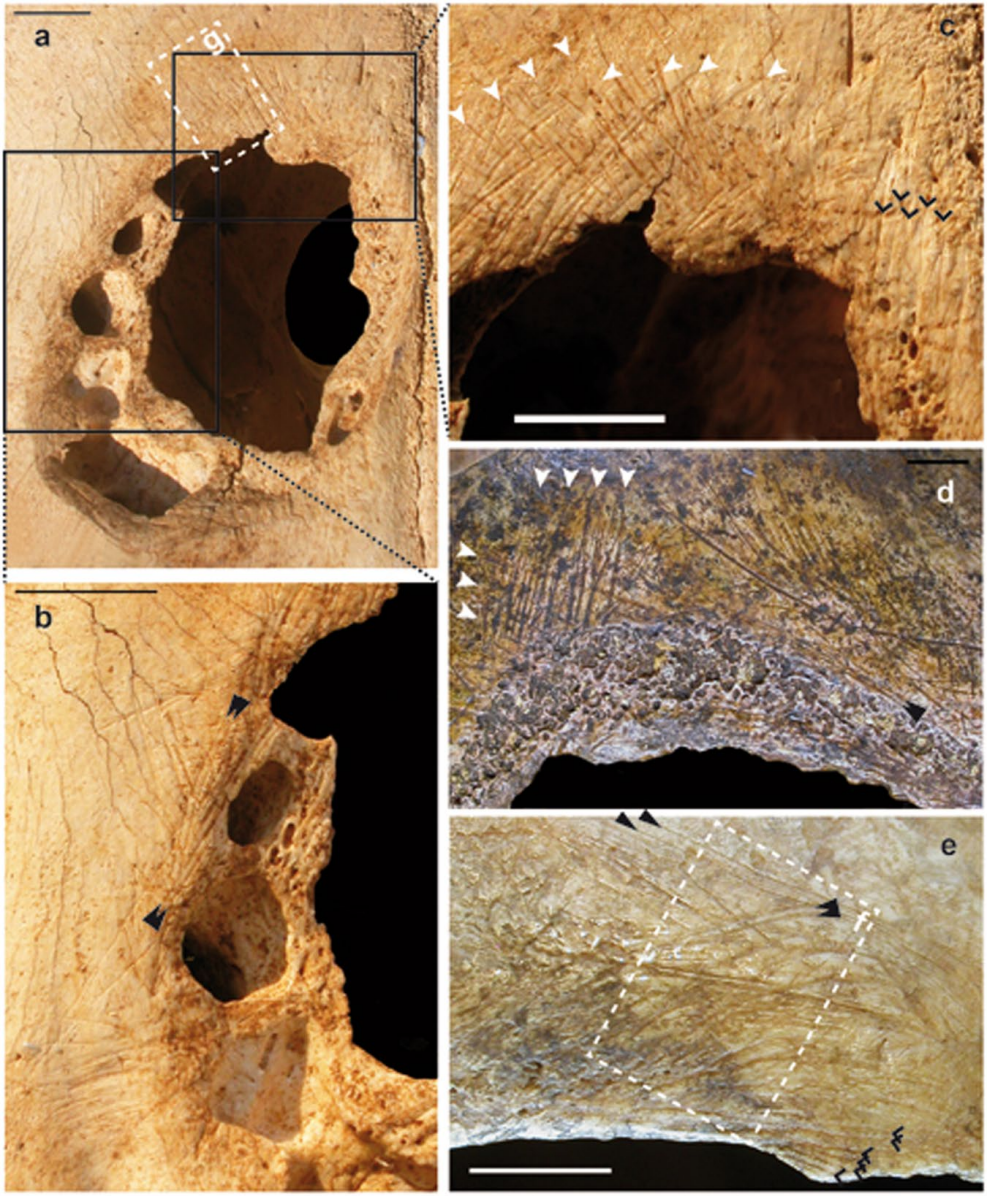
Cranial surgery in cow (**a**–**c**) compared with two human crania from the Neolithic period in France ((**d**) [28217bis], (**e**) [17144]). The cranial surgery in the cow cranium does not appear different to cranial surgery practiced on human crania. The use of a low magnification approach with either hand lenses or binoculars is more practical for identifying complete assemblages of cut-marks than a scanning electronic microscope^27^. More of the cut-marks appear in groups crossing between them and are obliquely orientated to the border of the perforation (white arrows). Parallel long cut-marks produced by a single tool in a unique gesture can be seen in cow as in human crania (black arrows). Other cut-marks with similar orientation show a large space between them and are almost parallel to the border of perforation; these cut-marks are probably associated with cutting more than with grasping (chevrons). Bar corresponds to 1 cm.

**Figure 3:**
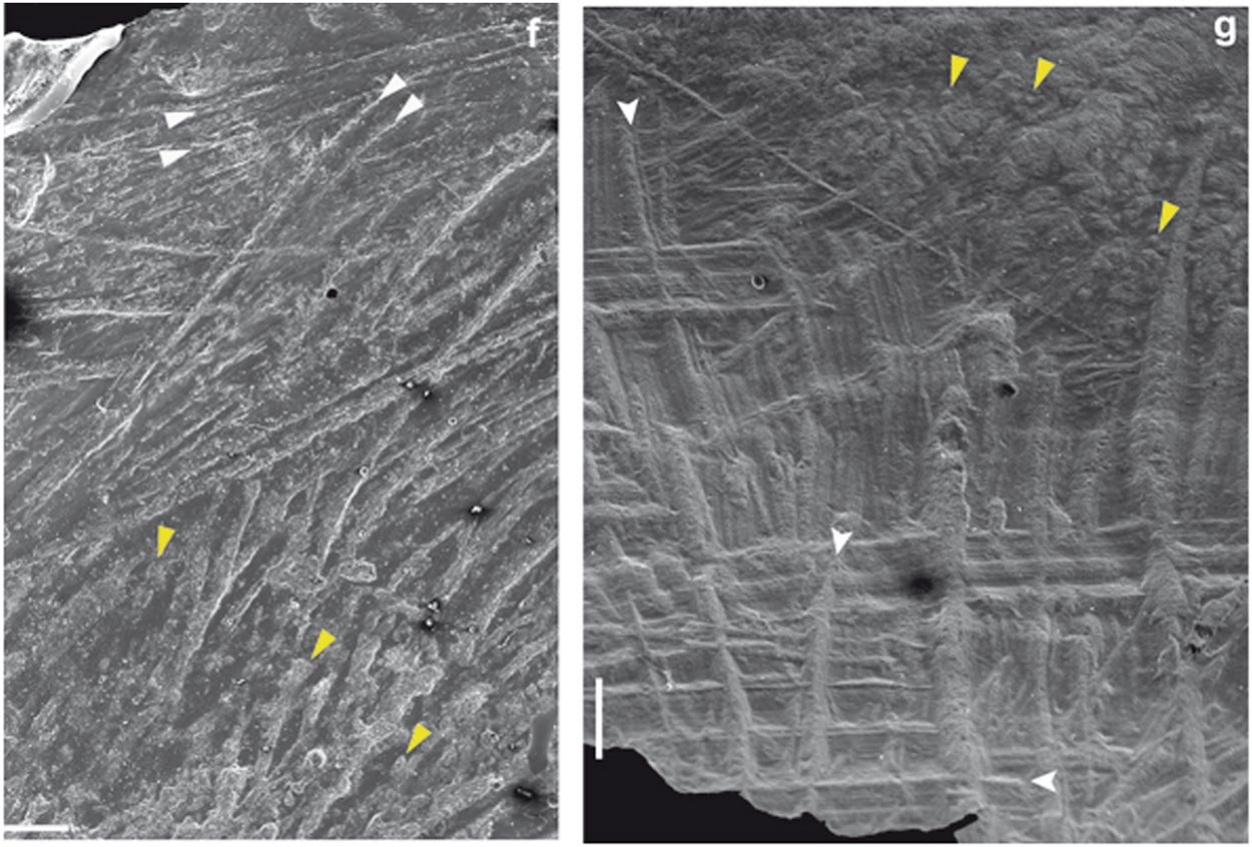
The SEM image of the detail of trepanation in a human cranium ((**f**) [17144]) enables to distinguish characteristic long, straight, multiple parallel cut-marks (white arrows) from short, irregular and rounded orifices of vascular channels (yellow arrows) predominant in the lower part of the picture. In the cow cranium (**g**), cut marks (white arrows) appear with their typical aspect: straight, multiple parallel, v-shaped and with micro-striations along the groove^27^, close to the border of the cranial surgery, whereas vascular channels are visible far from it (yellow arrows). Orientation, aspect, and packing of cut-marks reveal the same gestures in the crania analyzed, thus suggesting that the technique used on the cow cranium was the same as that used on human crania. Bar corresponds to 1 mm.

Marks consistent with any form of scraping are apparent around the hole. Indeed, in the posterior border, groups of cut-marks showing a different orientation suggest that the bone was intensively scraped (Figs 2a–c and 3g). In the anterior border, cut-marks are also observed but with a lesser density. Therefore, the almost square appearance of the hole, the lack of any mark indicating pressure exerted by an exterior force, the lack of any defects of the cranial vault associated with any illness and the presence of cut-marks all around the hole suggest that the injury derived from some form of surgical procedure typical of the trepanation process. The type and density of cut-marks on the cow cranium are similar to those observed in human skulls following cranial surgery by scraping (Figs 2d,e and 3f, see i.e. ref.^5^ Fig. 10.3, ref.^10^, Figs 5 and 7). Indeed, scraping was used to expose soft tissues in the cow, similar to the technique recorded for humans.

## Discussion

Bone remodeling following an injury starts several days later^24^. The lack of healing at histological level means that cranial surgery would have been practiced at a pre-mortem or a peri-mortem stage without the survival of the individual or alternatively that it occurred when the animal was already dead (post-mortem stage).

If cranial surgery on the cow employing the same techniques used on humans was indeed practiced in a pre-mortem or peri-mortem stage, it can be argued that the surgical intervention was carried out in order to save the animal. However, no abnormality or symptom of illness is observed in the cranium. Trauma is the most common cause for cranial surgery in some regions^10^. In a previous rapid survey of faunal remains on this site, it was suggested that goring by the horn of another animal caused the hole in the cow’s cranium^23^. However, there is no fracture near the trepanation or any indication of a shock to the cranium (Supplementary Fig. 1). Cranial surgery could remove any evidence of trauma^25^, but evidence of trauma most often disappears by healing^26^. Surgical intervention probably followed seizures or epilepsy or some other alteration of behavior. If such an intervention was carried out for the survival of the animal, it is worth noting that as early as the Neolithic period, these kinds of symptoms were already linked to brain physiology and/or activity revealing that a particular disorder in behavior was directly related to brain function. However, it is not clear what the interest would be in saving an animal of a species that was most commonly consumed for food. Archeological evidence reveals intensive economic activity around cattle and discards the notion that the cow was part of a ritual practice. If the Neolithic surgeon was not just practicing on the cow, or acting to treat it, removing a roundel for making an amulet, or performing a magical ritual, is of course unclear, but it is reasonable to suppose that any of those actions would have had greater value, practical or symbolic, if performed on a human being rather than on a common animal.

Cranial surgery may have been carried out when the cow was dead. Trepanation in this case would suggest that Neolithic man honed the techniques of cranial surgery on domestic animals before treating and caring for humans. Indeed, cranial surgery requires great manual dexterity and a complete knowledge of the anatomy of the brain and vessel distribution. It is possible that the mastery of techniques in cranial surgery shown in the Mesolithic and Neolithic periods was acquired through experimentation on animals. Cranial surgery as a practice could also have been performed on live animals; the lack of healing in the cow analyzed here could reveal a failure of the surgical intervention.

In conclusion, if cranial surgery observed on the cow was performed in order to save the animal, Champ-Durant provides the earliest evidence of veterinary surgical practice. Alternatively, if trepanation was used to practice techniques, the cow from Champ-Durand would provide the earliest evidence of surgical experimentation on an animal indicating that this practice already existed in 4000 BC.

## Materials and Methods

In order to evaluate the nature of the hole in the cow cranium, direct observation of the bone surface was performed with a stereomicroscope Wild M8 coupled with a Spot Idea camera. High-quality epoxy resin replicas of the bone close to the trepanation were obtained from hydrophobic vinyl polysiloxane (Coltène®) impressions. Replicas were later covered with gold-palladium to be observed under the SEM. In addition, a 3D X-scan of the whole cranium was obtained to assess damage to the bone and close-up radiographs were taken around the hole with a NOMAD, a handheld x-ray machine (Aribex), coupled with a digital x-ray sensor RSV2 (Visiodent). Similar analyses were performed on eighteen trepanned human crania from French Neolithic sites (Table 1).

**Table 1:** Human skulls with trepanation from French Neolithic sites housed at Musée de l’Homme used to compare the cow cranium.

NUMBER	TYPE	REGION	LOCALITY	SITE
12471	parietal	Oise	Belle-Haie	dolmen of Belle-Haie
17144	cranium	Lozère	Saint-Pierre-des-Tripiez	cave of Homme Mort
17148	cranium, fragment	Lozère	Aiguières	dolmen of Aiguières
17176	cranium	Lozère	Roussec	dolmen of Roussec
17352	cranium, fragment	Lozère	Aiguières	dolmen of Aiguières
17356	cranium, fragment	Lozère	Aiguières	dolmen of Aiguières
17357	cranium, fragment	Lozère	Aiguières	dolmen of Aiguières
17363	piece from trepanation	Lozère		
20973	cranium	Marne	Petit Morin	
24442	cranium	Lozère	Saint-Pierre-des-Tripiez	cave of Homme Mort
24901	calvaria	Yvelines	Les Mureaux	dolmen of Mureaux
25264-1	cranium, fragment	Yvelines	Les Mureaux	dolmen of Mureaux
25265	cranium, fragment	Yvelines	Les Mureaux	dolmen of Mureaux
25266	piece from trepanation	Yvelines	Les Mureaux	dolmen of Mureaux
28217	cranium	Seine-et-Marne	Vendrest	dolmen of Belleville
28217-bis	cranium	Seine-et-Marne	Vendrest	dolmen of Belleville
28816	cranium	Oise	Feigneux	
34956	calvaria	Val-d’Oise	Ménouville	

## Electronic supplementary material

Supplementary Information: Archaeological background
