# Supplementary material for: Earliest Animal Cranial Surgery: from Cow to Man in the Neolithic
Source: Sci Rep. 2018 Apr 19;8:5536. doi: 10.1038/s41598-018-23914-1 (PMC5908843; doi:10.1038/s41598-018-23914-1)

Earliest Animal Cranial Surgery: from Cow to Man in the Neolithic

Fernando Ramirez Rozzi[1*], Faculté de Chirurgie Dentaire, 1 rue Maurice Arnoux, 92120 Montrouge, France. +33 664006179. ramrozzi@yahoo.fr, fernando.ramirez-rozzi@cnrs.fr

Alain Froment[2], IRD - Musée de l'Homme, 17 place du Trocadéro 75116 Paris, France

Supplementary Information

Archaeological background

The archaeological site of Champ-Durand, in the Vendée area, Western France, discovered in 1971 by aerial prospection, consists of three concentric surrounding walls enclosing a space of three hectares. Before each surrounding wall, a system of ditches with enclosures reinforced the protection of the site. Archaeological excavations of the ditches and some other specific areas were conducted from 1975 to 1988 under the supervision of R. Joussaume (22).

The stratigraphy of the ditches shows a thin level at the bottom made of fine particles corresponding to a process of erosion by atmospheric agents, with big calcareous rocks in the center of the ditch originally part of the sidewalls. This first level corresponds to the period when the ditches were dug, and the outside wall designed to protect the site was built with the rocks extracted from the ditches. It is covered by a thick level composed of detrital discharges admixed with calcareous rocks coming from the sidewalls of the ditch. This level corresponds to the main occupation of Champ-Durand during the Final Neolithic. The remains of the collapsed wall on top marks the end of the occupation. In an upper level, some remains from another culture indicate a later occupation of the site, during a posterior phase of the Final Neolithic. It is followed by some Chalcolithic evidence at a time when the ditches had almost completely disappeared.

Most of the archaeological material was found between the bottom of the ditches and the level of the collapsed walls. Human remains are present, either as complete skeletons buried inside the ditches, or as scattered isolated bones. Regarding the 7 complete skeletons, the bone

conservation (low fragmentation, some elements in anatomic connection) indicate that they were buried on the slope of the ditches under the sidewalls. There is no evidence of decay, suggesting that the bodies were protected to some extent. Burying human bodies in ditches was a current practice during the Neolithic in Europe (29, 30). Skeletons are isolated or grouped by twos but in that case it is impossible to say if the burials were simultaneous or serial. Beside human remains were found parts of domesticated animals typical of the Neolithic period (bovines, caprinae [sheep and goats], pigs) but also dogs and wild animals such as deer and boars. Whereas skeletons were located inside the ditches of the intermediary wall and the southern wall, 32 fragmented human bones were found between the bottom of the ditches and the level of collapsed walls of all three ditches: internal, intermediary and external. Most of these 32 remains belong to skulls or mandibles; others are large fragments which could not penetrate by infiltration; some could belong to the same individuals, later disrupted. Many dog bones have been found in the vicinity of human skeletons, which suggests a significant association in these deposits, a fact already noted elsewhere in the region. Some human skulls, either isolated or found in anatomical connection, display evidence of traumas which could have been lethal. An isolated child's skull bears three incisions on the posterior part of the right parietal, but none of the skulls underwent trepanation.

Faunal remnants are abundant, over 44,000 pieces of bones, of which 65% could be identified. Oxen are the most represented animals, with more than 50% of the total, pigs and caprinae each represent about 20%. Dogs, the other domesticated animal found at Champ-Durand, correspond only to 2%. Deer, boars and other game animals are each respectively represented by less than 1% of total fauna. Skulls, mandibles and isolated teeth, as well as vertebrae, are very frequent among domesticated species. A calculation of the minimum number of individuals gives 95 oxen, 59 caprinae and 35 pigs. All classes of age are represented but most bones correspond to young subjects. A very small percentage of these three groups of

animals display cutting marks or burns. Among these remains, the trephined cow skull is the only one to show specific treatment. This skull was found in the intermediary wall (FII) in sector 80, in the same sediment as all other faunal remains in the area. The study of animal remains suggests that the main species bred at Champ-Durand were bovines, followed by caprinae and pigs as a complement. Other contemporary sites in the area display the same pattern. The abundance of bones coming mainly from the axial skeleton and legs, as well as the low percentage of pieces bearing evidence of dietary activity, suggest primary butchery.

Twenty-five AMS 14C dates have been determined on skulls or fragments of skulls found in the ditches, 19 of human origin and 6 from animals (31). All these remains have been found between the bottom of the ditches and the level sealed by the collapse of the walls. All the dates fall within 3500 and 3000 BC. Regarding the area of the intermediary wall (FII) where the cow's skull was discovered, the four dates obtained vary between 3028 and 3485 BC, which fits well inside the chronological range of the entire site. The age of the cow's skull can then clearly be situated between 3400 and 3000 BC.

All the studies of this archaeological site suggest that Champ-Durand was a fortified village of farmers/cultivators living in the Final Neolithic, between 3400 and 3000 BC. The principal aim of such a site was to protect cattle from various predators. This type of village was abundant in this region at that period. The group inhabiting this site had a specific regional culture, as revealed by their pottery, called Seuil du Poitou, related to the Peu-Richardien civilization living near the coast in Western France at that time.

References Supplementary Information

29 Pariat, J.-G. *Des morts sans tombe ? Le cas des ossements humains en contexte non sépulcral en Europe tempérée entre les 6e et 3e millénaires av. J.-C*. BAR International Series, 1683, 195 p. (2007).

30 Soler, L. Les dépôts humains du littoral charentais. In *Des premiers paysans aux premiers métallurgistes sur la façade atlantique de la France (3500-200 av. J.-C.),* (Laporte, L. ed.) Ed. Association des Publications Chauvinoises, Mémoire XXXIII, Chauvigny, p. 647-655 (2009).

31 Ard, V., Large, J.-M. & Soler L. Nouvelles datations au radiocarbone pour le site de Champ-Durand à Nieul-sur-l'Autise (Vendée). In *L'enceinte Néolithique de Champ-Durand à Nieul-sur-l'Autise (Vendée)* (Jaussaume, R. ed.) Mémoire XLIV, Conseil Régional Vendée, p. 643-652 (2012).

Supplementary Figure 1

Figure S1: SEM image of the interior face of the inner tabula of a part of the upper border of the trepanation. Bone shows no evidence of internally orientated cracks or splinters but shows a continuous surface of endocranium.

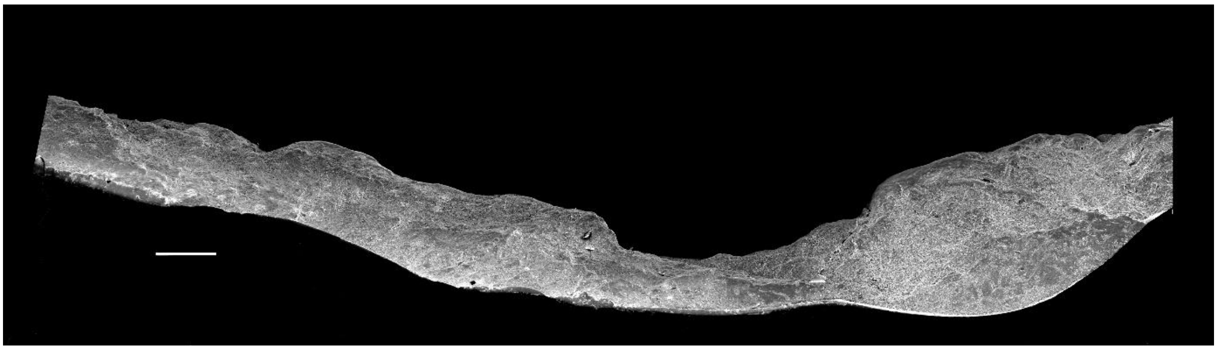

Supplement: Supplementary file 1 — Supplementary Information: Archaeological background [file 41598_2018_23914_MOESM1_ESM.pdf]
